# Cell-surface milieu remodeling in human dendritic cell activation

**DOI:** 10.4049/jimmunol.2400089

**Published:** 2024-10-01

**Authors:** Namrata D. Udeshi, Charles Xu, Zuzhi Jiang, Shihong Max Gao, Qian Yin, Wei Luo, Steven A. Carr, Mark M. Davis, Jiefu Li

**Affiliations:** * The Broad Institute of MIT and Harvard, Cambridge, MA 02142, USA; † Janelia Research Campus, Howard Hughes Medical Institute, Ashburn, VA 20147, USA; ‡ Yuanpei College, Peking University, Beijing 100871, China; § Institute of Immunity, Transplantation and Infection, Stanford University School of Medicine, Stanford, CA 94305, USA; ¶ Department of Biomedical Engineering, University of Texas at Austin, Austin, TX 78712, USA; ∥ Department of Microbiology and Immunology, Indiana University School of Medicine, Indianapolis, IN 46202, USA; # Howard Hughes Medical Institute, Stanford University, Stanford, CA 94305, USA

**Keywords:** proteomics, mass spectrometry, cell-surface proteome, toll-like receptor

## Abstract

Dendritic cells (DCs) are specialized sentinel and antigen presenting cells coordinating innate and adaptive immunity. Through proteins on their cell surface, DCs sense changes in the environment, internalize pathogens, present processed antigens, and communicate with other immune cells. By combining chemical labeling and quantitative mass spectrometry, we systematically profiled and compared the cell-surface proteomes of human primary conventional DCs (cDCs) in their resting and activated states. Toll-like receptor activation by a lipopeptide globally reshaped the cell-surface proteome of cDCs, with more than one hundred proteins up or down regulated. By simultaneously elevating positive regulators and reducing inhibitory signals across multiple protein families, the remodeling creates a cell-surface milieu promoting immune responses. Still, cDCs maintain the stimulatory-to-inhibitory balance by leveraging a distinct set of inhibitory molecules. This analysis thus uncovers the molecular complexity and plasticity of the cDC cell surface and provides a roadmap for understanding cDC activation and signaling.

## INTRODUCTION

Communication and coordination between the innate and adaptive immune systems is crucial for the defense against pathogens as well as the detection of cancerous cells. As professional antigen processing and presenting cells, dendritic cells (DCs) are at the center stage for innate-adaptive communication (1-6). DCs express many innate immune receptors to monitor the tissue environment, with a large fraction on the cell surface directly surveilling the extracellular space. When pathogens are detected, DCs quickly activate from a resting state, present antigens to T lymphocytes through cell-surface major histocompatibility complexes, and modulate the activities of many other leukocytes via intercellular ligand-receptor pairs, illustrating the fundamental roles of cell-surface proteins in diverse aspects of DC physiology. Although several cell-surface markers of DC activation are known, such as CD40, CD86, and HLAs, it is elusive whether DC activation leverages only a few select cell-surface proteins or globally remodels the DC cell-surface proteome. In either case, what is the complete repertoire of activation-regulated cell-surface proteins?

RNA sequencing-based transcriptomic analyses (7-9) have produced substantial insights into DC heterogeneity and identified subtype-specific molecular signatures; however, quantitative inferences of proteome dynamics from transcriptomic measurements are not ideal, particularly for cell-surface proteins due to their complicated trafficking, modification, and turnover control (10-15). High-dimensional cytometry (16, 17) measures proteins but only covers a select panel with available antibodies. Instead, mass spectrometry-based cell-surface proteomics offers a method for directly profiling cell-surface protein composition with proteome-wide coverage and quantification (11, 15, 18-23).

**Figure 1.**
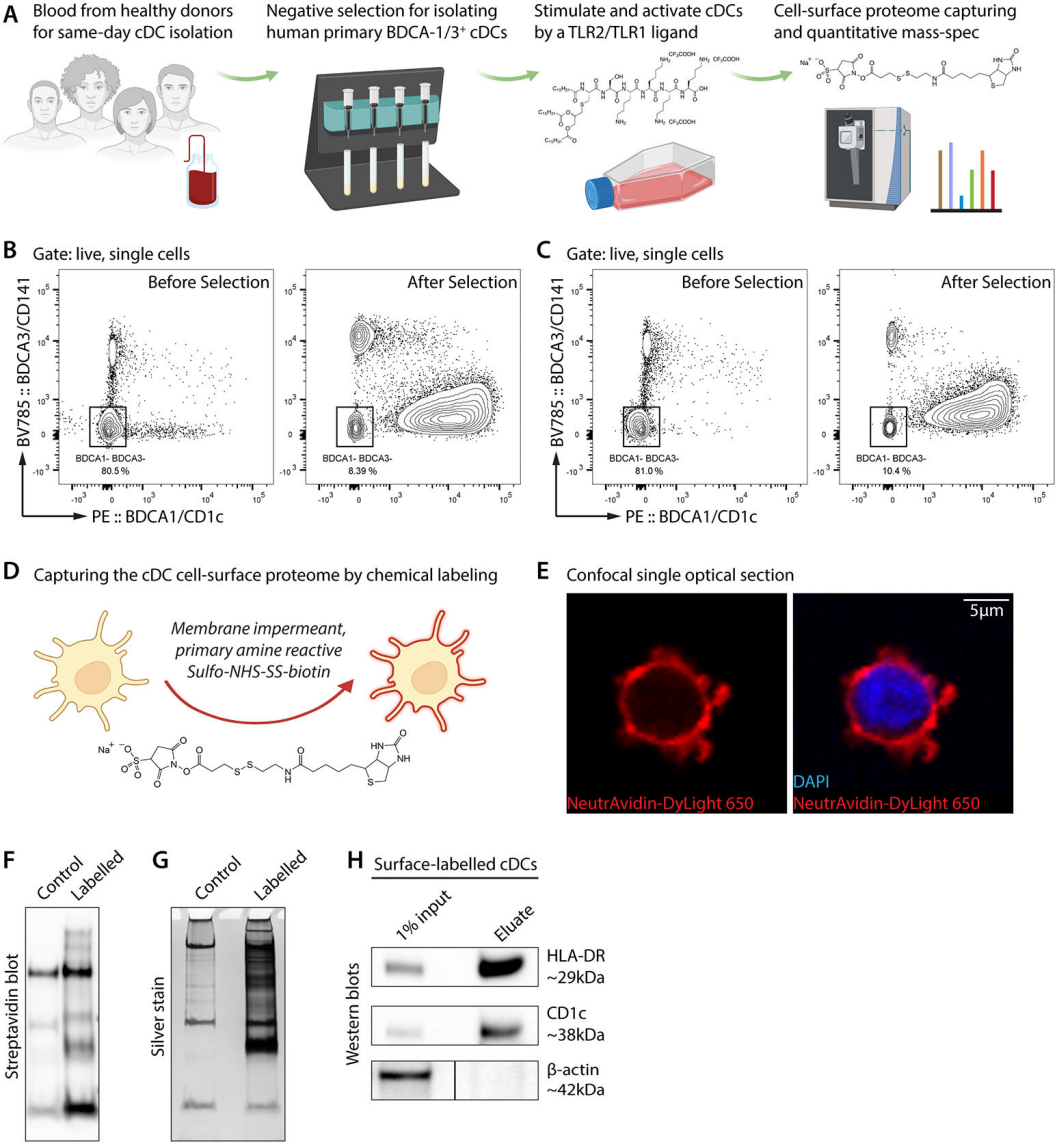
Chemical labeling and capturing of the cell-surface proteome of human primary conventional DCs. (**A**) Workflow of this study. Fresh buffy coats were obtained from healthy donors for same-day processing. Primary BDCA-1/3^+^ conventional DCs (cDCs) were isolated by magnetic negative selection and stimulated by the TLR2/TLR1 ligand Pam3CSK4 (0.5 μg/mL; chemical structure in the 3^rd^ panel) for 18 hours. Cell-surface proteins were labelled by Sulfo-NHS-SS-biotin (chemical structure in the 4^th^ panel) and analyzed by quantitative mass spectrometry. (**B** and **C**) Flow cytometry data, of two blood samples, validating the isolation of BDCA-1+ or BDCA-3+ cDCs through magnetic negative selection. Left, before selection; right, after selection. We note that negative selection minimally perturbs the targeted cDCs but slightly compromises the purity (~10% contamination of BDCA-1– and BDCA-3– cells). (**D**) Sulfo-NHS-SS-biotin is impermeant to the plasma membrane and reactive to primary amines. Thus, it covalently and specifically labels extracellular residues of cell-surface proteins and enables streptavidin-based protein enrichment. (**E**) A confocal optical section of a cell-surface biotinylated cDC. NeutrAvidin, for staining biotin. DAPI, 4’,6-diamidino-2-phenylindole for staining the nucleus. Scale bar, 5μm. (**F** and **G**) Streptavidin blot (**F**) and silver stain (**G**) showing protein enrichment in the cell-surface biotinylation group (labelled) but not the non-biotinylation group (control). Bands in the control lanes correspond to endogenously biotinylated proteins. (**H**) Western blots showing the specific enrichment of cDC cell-surface proteins HLA-DR and CD1c but not the intracellular protein β-actin.

To determine how activation changes the cell-surface proteomic milieu of human primary DCs, we isolated BDCA-1/3^+^ conventional DCs (cDCs) from healthy blood donors and stimulated them with a triacylated lipopeptide Pam3CSK4—a ligand for the Toll-like receptor (TLR) hetero-complex TLR2/TLR1 (Fig. 1A). Pam3CSK4 mimics the amino terminus of bacterial lipopeptides that are pro-inflammatory from both Gram-positive and Gram-negative bacteria. Quantitative cell-surface proteomics found that, within only 18 hours of TLR2/TLR1 activation, the cDC cell surface was globally remodeled into a pro-inflammatory environment. The remodeling program is highly coordinated and also hedged to boost immunity while retaining inhibitory signals.

## MATERIALS AND METHODS

### Human blood sample collection

The use of human blood samples and its protocols were approved by Institutional Review Boards of Stanford University and Janelia Research Campus, Howard Hughes Medical Institute. Buffy coats from healthy donors were obtained from the Stanford Blood Center on the same day of blood drawing and were processed immediately to isolate primary cDCs without refrigeration or freezing.

### Primary cDC isolation

Peripheral blood mononuclear cells (PBMCs) were isolated from buffy coats by centrifugation in the Ficoll-Paque density gradient media (Cytiva, 17144002). BDCA-1^+^ or BDCA-3^+^ conventional DCs were then isolated from PBMCs by magnetic negative selection using the Human Myeloid Dendritic Cell Isolation Kit (Miltenyi Biotec, 130-094-487) following the manufacturer’s protocol. Briefly, PBMCs were suspended in FACS buffer containing 0.5% bovine serum albumin (Sigma, A8806) and 2 mM ethylenediaminetetraacetic acid (ThermoFisher, 15575020) in phosphate buffered saline (Lonza, 17516F). 100 μL of FcR blocking reagent, 100 μL of non-myeloid dendritic cell antibody-biotin cocktail, and 100 μL of anti-biotin MicroBeads were used per 5×10^7^ total cells. LD columns and MACS magnets were used for cell separation.

### Flow cytometry

PBMCs and cDCs were stained in FACS buffer for 30 minutes at 4°C with the following reagents: human TruStain FcX Fc receptor blocking solution (BioLegend, 422302; 1:20), live/dead aqua dead cell stain kit (Invitrogen, L34966; 1×), anti-human CD1c/BDCA1 antibody conjugated with PE (BD, 564900; 1:100), and anti-human CD141/BDCA3 antibody conjugated with BV786 (BD, 741006; 1:100). Cells were washed, resuspended in FACS buffer, and analyzed on a BD FACSymphony flow cytometer. Data was analyzed using the FlowJo software (BD).

### Cell culture

Isolated cDCs were cultured in the X-VIVO 15 hematopoietic culture media (Lonza, 04418Q), supplemented with 10% heat-inactivated human AB serum (Sigma, H3667, Lot SLCF0444). 2 mg/ml Pam3CSK4 (InvivoGen, vac-pms) stock solution was made in phosphate buffered saline (Lonza, 17516F). For TLR2/TLR1 activation, 0.5 μg/mL Pam3CSK4 was added for 18 hours. For control groups, an equal volume of phosphate buffered saline was added.

### Cell-surface biotinylation

250 μg/mL Sulfo-NHS-SS-biotin (ThermoFisher, A39258) was freshly prepared in phosphate buffered saline (Lonza, 17516F). Cells were washed with phosphate buffered saline and then resuspended in the Sulfo-NHS-SS-biotin solution for 10 minutes at room temperature. The reaction was quenched using ice-cooled Tris-buffered saline (ThermoFisher, 28379). Cells were washed twice using ice-cooled Tris-buffered saline before liquid nitrogen snap-freezing for biochemistry or paraformaldehyde fixation for fluorescence imaging.

### Confocal microscopy

Paraformaldehyde-fixed cells were membrane-permeabilized and blocked using 0.3% Triton X-100 (ThermoFisher, 85111) and 5% bovine serum albumin (Sigma, A8806) in phosphate buffered saline (Lonza, 17516F). Cells were then stained with 1 μg/mL NeutrAvidin conjugated with DyLight 650 (ThermoFisher, 84607) for 30 minutes at room temperature in 0.015% Triton X-100 and 5% bovine serum albumin in phosphate buffered saline. Cells were washed three times using 0.015% Triton X-100 in phosphate buffered saline and mounted in an antifade media containing 4′,6-diamidino-2-phenylindole for nucleus staining (ThermoFisher, P36981). Fluorescence images were acquired with a Zeiss LSM 780 laser-scanning confocal microscope using a 40x oil objective. Single optical sections were acquired at 1-mm intervals at the resolution of 512×512. Images were analyzed using the Zen software (Zeiss).

### Cell lysis

Cells were lysed in the original collection tube (Eppendorf, 022431081) to minimize material loss during transfer. For each group (~7.4×10^6^ cells), the cell pellet was resuspended in 150 μL high-SDS RIPA buffer (Sigma, R0278) containing 1% sodium dodecyl sulfate (SDS; Sigma, L6026) and 1× protease inhibitor cocktail (ThermoFisher, 78438) and lysed by pipetting and vortexing. 1200 μL low-SDS RIPA buffer containing 0.1% SDS was then added to bring down the SDS concentration to 0.2%. Lysates were rotated at 4°C for 2 hours before centrifugation at 30,000 g and 4°C for 30 minutes. Supernatants were collected.

### Streptavidin bead enrichment

Streptavidin magnetic beads (ThermoFisher, 88817) were used to enrich biotinylated proteins from cell lysates. For each group (lysate from ~7.4×10^6^ cells), 300 μL of streptavidin beads was washed twice with 1 mL 0.2%-SDS RIPA buffer (Sigma, R0278 and L6026) and then incubated with the post-centrifugation lysates on a 4°C rotator overnight. Beads were then sequentially washed twice with 1 mL 0.2%-SDS RIPA buffer, once with 1 mL 1 M KCl (Sigma, P9333), once with 1 mL 0.1 M Na_2_CO_3_ (Sigma, S7795), once with 1 mL 2 M urea (Sigma, 51456) in 10 mM Tris-HCl [pH 8.0] (ThermoFisher, 15568025), and twice with 1 mL 0.2%-SDS RIPA buffer. 1× protease inhibitor cocktail (ThermoFisher, 78438) was added to all solutions used above. Beads were then washed twice with 1 mL phosphate buffered saline without the protease inhibitor cocktail before on-bead trypsin digestion or protein blotting (see below for details).

### Protein blotting and silver stain

Biotinylated proteins were eluted by heating streptavidin beads at 95°C for 10 minutes in 2× LDS sample buffer (ThermoFisher, B0007) containing 1× reducing agent (ThermoFisher, B0009) and 2 mM biotin (Sigma, B4501). 4%–12% Bis-Tris PAGE gels (ThermoFisher, NW04120BOX) were used for protein electrophoresis. A silver stain kit (ThermoFisher, 24612) was used for total protein detection following the manufacturer’s protocol. For protein blotting, proteins were transferred to PVDF membranes (ThermoFisher, IB24001). After blocking (ThermoFisher, 37536) at room temperature for 1 hour, membranes were incubated with primary antibodies diluted in the blocking buffer on a 4°C orbital shaker overnight. Following four rounds of washes in Tris-buffered saline with Tween 20 (ThermoFisher, 28360), membranes were incubated with horseradish peroxidase (HRP)-conjugated secondary antibodies diluted in the blocking buffer at room temperature for 1 hour and then washed four rounds in Tris-buffered saline with Tween 20. Clarity Western ECL substrate (BioRad, 1705060) and a ChemiDoc system (BioRad) were used for chemiluminescence development and detection.

Antibodies used for protein biochemistry included: rabbit anti-human HLA-DR (Abcam, ab92511; 1:2,500), rabbit anti-human CD1c (Abcam, ab246520; 1:1,000), mouse anti-human β-actin (Abcam, ab8224; 1:1,000), goat anti-rabbit IgG conjugated with HRP (Abcam, ab6721; 1:3,000), and goat anti-mouse IgG conjugated with HRP (Abcam, ab97023; 1:3,000). Streptavidin conjugated with HRP (ThermoFisher, N100; 1:3,000) was used to detect biotinylated proteins.

### On-bead trypsin digestion

Samples collected and enriched with streptavidin magnetic beads were washed twice with 200 μL of 50 mM Tris-HCl buffer (pH 7.5), transferred into new 1.5 mL Eppendorf tubes, and washed 2 more times with 200 μL of 50 mM Tris (pH 7.5) buffer. Samples were incubated in 0.4 μg of trypsin in 80 μL of 2 M urea/50 mM Tris buffer with 1 mM dithiothreitol (DTT), for 1 hour at room temperature while shaking at 1000 rpm. Following pre-digestion, 80 μL of each supernatant was transferred into new tubes. Beads were then incubated in 80 μL of the same digestion buffer for 30 minutes while shaking at 1000 rpm. Supernatant was transferred to the tube containing the previous elution. Beads were washed twice with 60 μL of 2 M urea/50 mM Tris buffer, and these washes were combined with the supernatant. The eluates were spun down at 5000 ×g for 30 seconds and the supernatant was transferred to a new tube. Samples were reduced with 4 mM DTT for 30 minutes at room temperature, with shaking. Following reduction, samples were alkylated with 10 mM iodoacetamide for 45 min in the dark at room temperature. An additional 0.5 μg of trypsin was added and samples were digested overnight at room temperature while shaking at 700 rpm. Following overnight digestion, samples were acidified (pH<3) with neat formic acid (FA), to a final concentration of 1% FA. Samples were spun down and desalted on C18 StageTips as previously described. Eluted peptides were dried to completion and stored at −80°C.

### TMT labeling and StageTip peptide fractionation

Desalted peptides were labelled with TMT (6-plex) reagents (ThermoFisher). Peptides were resuspended in 80 μL of 50 mM HEPES and labelled with 20 μL of 20 mg/mL TMTpro18 reagents in acetonitrile (ACN). Samples were incubated at room temperature for 1 hour with shaking at 1000 rpm. TMT reaction was quenched with 4 μL of 5% hydroxylamine at room temperature for 15 minutes with shaking. TMT-labelled samples were combined, dried to completion, reconstituted in 100 μL of 0.1% FA, and desalted on StageTips.

The TMT-labelled peptide sample was fractionated by basic reverse phase (bRP) fractionation. StageTips were packed with two disks of SDB-RPS (Empore) material and conditioned with 100 μL of 100% MeOH, followed by 100 μL of 50% MeCN/0.1% FA and two washes with 100 μL of 0.1% FA. Peptide samples were resuspended in 200 μL of 1% FA (pH<3) and loaded onto StageTips. 6 step-wise elutions were carried out in 100 μL of 20 mM ammonium formate buffer with increasing concentration of 5%, 10%, 15%, 20%, 25%, and 45% MeCN. Eluted fractions were dried to completion.

### Liquid chromatography tandem mass spectrometry

Online separation was done with a nanoflow Proxeon EASY-nLC 1200 UHPLC system (ThermoFisher). In this set up, the LC system, column, and platinum wire used to deliver electrospray source voltage were connected via a stainless steel cross (360 mm, IDEX Health & Science, UH-906x). The column was heated to 50°C using a column heater sleeve (Phoenix-ST). Each sample was injected onto an in-house packed 27 cm x 75um internal diameter C18 silica picofrit capillary column (1.9 μm ReproSil-Pur C18-AQ beads, Dr. Maisch GmbH, r119.aq; Picofrit 10 um tip opening, New Objective, PF360-75-10-N-5). Mobile phase flow rate was 200 nL/min, comprised of 3% acetonitrile/0.1% formic acid (Solvent A) and 90% acetonitrile/0.1% formic acid (Solvent B). The 154-min LC-MS/MS method used the following gradient profile: (min:%B) 0:2;2:6; 122:35; 130:60; 133:90; 143:90; 144:50; 154:50 (the last two steps at 500 nL/min flow rate). Data acquisition was done on an Exploris mass spectrometer (ThermoFisher) in the data-dependent mode acquiring HCD MS/MS scans (r = 15,000) after each MS1 scan (r = 60,000) on the top 12 most abundant ions using a normalized MS1 AGC target of 100% and an MS2 AGC target of 50%. The maximum ion time utilized for MS/MS scans was 120 ms; the HCD-normalized collision energy was set to 34; the dynamic exclusion time was set to 20 s, and the peptide match and isotope exclusion functions were enabled. Charge exclusion was enabled for charge states that were unassigned, 1 and >6.

### Mass spectrometry data processing

Mass spectrometry data was processed using Spectrum Mill v7.11. For all samples, extraction of raw files retained spectra within a precursor mass range of 600-6000 Da and a minimum MS1 signal-to-noise ratio of 25. MS1 spectra within a retention time range of +/− 45 s, or within a precursor m/z tolerance of +/− 1.4 m/z were merged. MS/MS searching was performed against a human UniProt database with a release date of December 28, 2017. Digestion parameters were set to “trypsin allow P” with an allowance of 4 missed cleavages. The MS/MS search included fixed modification of carbamidomethylation on cysteine. TMT6 was searched using the partial-mix function. Variable modifications were acetylation, CAMthipropanoylation, and oxidation of methionine. Restrictions for matching included a minimum matched peak intensity of 30% and a precursor and product mass tolerance of +/− 20 ppm.

Peptide spectrum matches were validated using a maximum false discovery rate (FDR) threshold of 1.2% for precursor charges 2 through 6 within each LC-MS/MS run. TMT reporter ion intensities were corrected for isotopic impurities in the Spectrum Mill protein/peptide summary module using the afRICA correction method which implements determinant calculations according to Cramer’s Rule. We required fully quantified unique human peptides and a protein score >9 for protein quantification. We used the Proteomics Toolset for Integrative Data Analysis (Protigy, v0.8.6.3, Broad Institute; https://github.com/broadinstitute/protigy) to calculate moderated *t*-test *p* values for regulated proteins.

We note that because of HLA protein similarity and polymorphism, unambiguously identifying and assigning HLA alleles with tryptic peptides without paired DNA sequencing is technically challenging (24). Due to the sparseness of primary DCs, we had to pool samples from multiple donors with different HLA profiles; therefore, the assignment and quantification of specific HLA alleles are subject to technical inaccuracy.

### Proteomic data analysis

Supplemental Table – Tab 2 contains SwissProt-curated mitochondrial (UniProt: SL-0173), nuclear (SL-0191), and cytosolic (SL-0091) proteins without extracellular (SL-0112), secreted (SL-0243), signal peptide-containing (KW-0732), type II transmembrane (SL-9906), or type III transmembrane (SL-9907) annotations. Among the 2,640 human proteins detected with at least 2 unique peptides, 1,175 were found in Supplemental Table – Tab 2 and thus filtered out as intracellular contaminants. Gene ontology analyses were performed using the Panther server. The BioGRID database and the Cytoscape software were used for protein-protein interaction analyses.

### Quantification and statistical analysis

Statistical tests are indicated in figure legends. Data collection and analysis were not performed blind to the conditions of the experiments. Excel (Microsoft) and Prism (GraphPad) were used for data analysis and plotting.

### Data availability

Raw mass spectrometry data have been deposited in the public proteomics repository MassIVE and are accessible at ftp://MSV000092573@massive.ucsd.edu using the password: proximity. If requested, also provide the username: MSV000092573. The data will be made publicly available upon acceptance of the manuscript. Proteomic results are provided in Supplemental Table – Tab 1-4.

## RESULTS

### Capturing the cell-surface proteome of human primary cDCs

Dendritic cells are sensitive to the environmental change and known to have a short life span (1, 25). To minimize proteomic alterations and artifacts caused by cell processing, we obtained fresh buffy coats from healthy donors and isolated BDCA-1/3^+^ cDCs by magnetic negative selection on the same day of blood collection (Fig. 1A, left two panels)—without refrigeration, freezing, or sorting—yielding highly-enriched cDCs (Fig. 1B, 1C).

We then used a chemical biotinylation strategy (18) to label and capture cDC cell-surface proteins (Fig. 1D). N-hydroxysulfosuccinimide (Sulfo-NHS) is plasma membrane-impermeable and primary amine-reactive. By non-selectively conjugating to the N-termini or lysine residues of extracellular polypeptides, this chemical covalently tags cell-surface proteins with biotin, enabling further detection and purification. NeutrAvidin staining, which recognizes biotin, showed that biotinylation was extensive on and exclusive to the cell surface (Fig. 1E). Biotinylated cell-surface proteins could be enriched by streptavidin beads, as illustrated by streptavidin blot and silver stain of bead eluate (Fig. 1F, 1G). Known cDC cell-surface proteins HLA-DR and CD1c were enriched (Fig. 1H, upper two panels) while the abundant intracellular protein β-actin was not (Fig. 1H, lower panel), further confirming the cell-surface specificity of this method.

### Quantitative mass spectrometry profiling of the cDC cell-surface proteome

**Figure 2.**
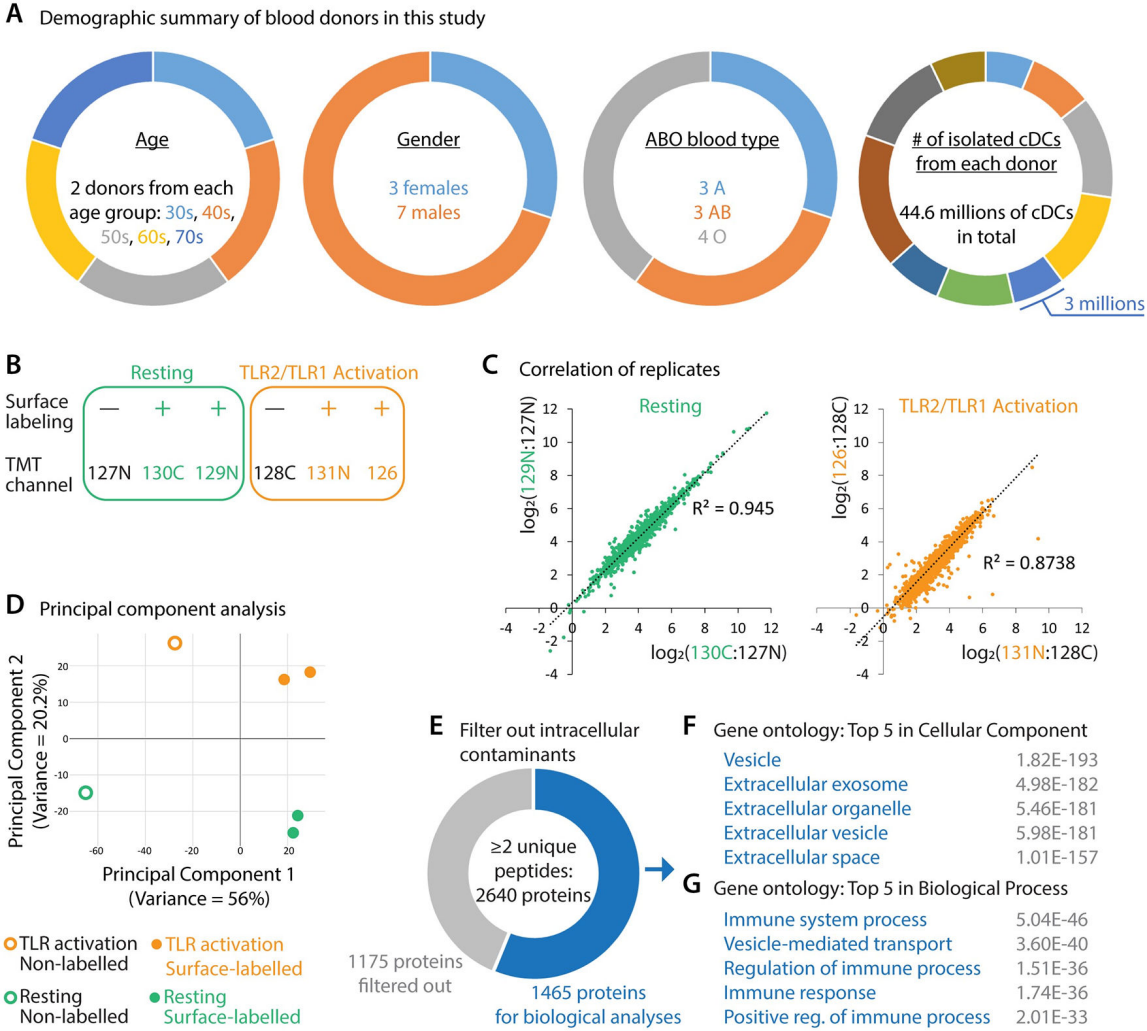
Cell-surface proteomic profiling of resting and TLR2/TLR1-activated human cDCs. (**A**) Demographic summary of blood donors in this study. (**B**) Design of the six-plex tandem mass tag (TMT)-based quantitative proteomic experiment. Each cDC state (green, resting; orange, TLR2/TLR1 activation) was profiled by two replicates with cell-surface biotinylation (+) and one non-biotinylation control (−). Labels in the TMT channel row indicate the TMT tag used for each condition. In total, this experiment identified 3,026 proteins, including 2,640 human proteins with at least two unique peptides detected. (**C**) Correlation plots of replicates. (**D**) Principal component analysis of all six conditions. Green circle, non-biotinylation control of resting cDCs (TMT 127N). Green dots, cell-surface biotinylation groups of resting cDCs (TMT 130C and 129N). Orange circle, non-biotinylation control of TLR2/TLR1-activated cDCs (TMT 128C). Orange dots, cell-surface biotinylation groups of TLR2/TLR1-activated cDCs (TMT 131N and 126). (**E**) 1,175 detected proteins (gray) were curated as mitochondrial, nuclear, or cytosolic proteins by Swiss-Prot and were therefore filtered out. 1,465 proteins were retained for biological analyses. (**F** and **G**) Top 5 gene ontology terms enriched for the post-filtering proteins (blue, **E**) regarding subcellular localization (**F**) and biological function (**G**). Gray numbers indicate the false discovery rate of each term.

Primary cDCs constitute a rare population in human peripheral blood mononuclear cells while cell-surface proteins account for a small fraction of total proteins in any given cells. Thus, the protein sample enriched from one donor is not sufficient for in-depth proteomic analysis. We therefore combined ten individual donors from diverse demographic backgrounds (Fig. 2A). To avoid artifacts caused by individual variability, isolated cDCs of each donor were evenly distributed into six groups (Fig. 2B): two replicates for TLR2/TLR1 activation by the lipopeptide Pam3CSK4 (orange, “+” indicating cell-surface biotinylation), two replicates for the resting state without Pam3CSK4 (green, “+”), and two negative samples without cell-surface biotinylation (black, “−”).

To quantitatively assess cDC cell-surface proteomic change upon TLR2/TLR1 activation, we used a six-plex tandem mass tag (TMT) strategy (26) and identified 2,640 proteins with at least two unique peptides (Supplemental Table – Tab 1). In both resting and TLR2/TLR1 activation groups, the replicates showed high correlations (Fig. 2C). Principal component analysis found that each pair of replicates clustered together and separated from other conditions (Fig. 2D), with the first two principal components corresponding to cell-surface biotinylation (x-axis, principal component 1) and TLR2/TLR1 activation (y-axis, principal component 2).

As in any enrichment or pull-down mass spectrometry analysis, we detected contaminant proteins—in this case of cell-surface profiling, intracellular proteins—caused by endogenous biotinylation in the mitochondrial respiratory chain, non-specific bead binding, and intracellular Sulfo-NHS labeling due to membrane rupture or cell death. To remove intracellular contaminants, we filtered out 1,175 proteins curated as nuclear, mitochondrial, or cytosolic proteins by the Swiss-Prot consortium (27) (Supplemental Table – Tab 2) and obtained 1,465 proteins for further analyses (Fig. 2E and Supplemental Table – Tab 3). Gene ontology analyses of these 1,465 proteins found that they were highly enriched for cell-surface and extracellular proteins (Fig. 2F) and functionally linked to immune processes (Fig. 2G). We note that, due to database annotation incompleteness, there were still intracellular contaminants in the post-filtering protein list (Supplemental Table – Tab 3), such as some ribosomal proteins (Supplemental Fig. 1).

### Cell-surface proteomic landscape of activated cDCs

**Figure 3.**
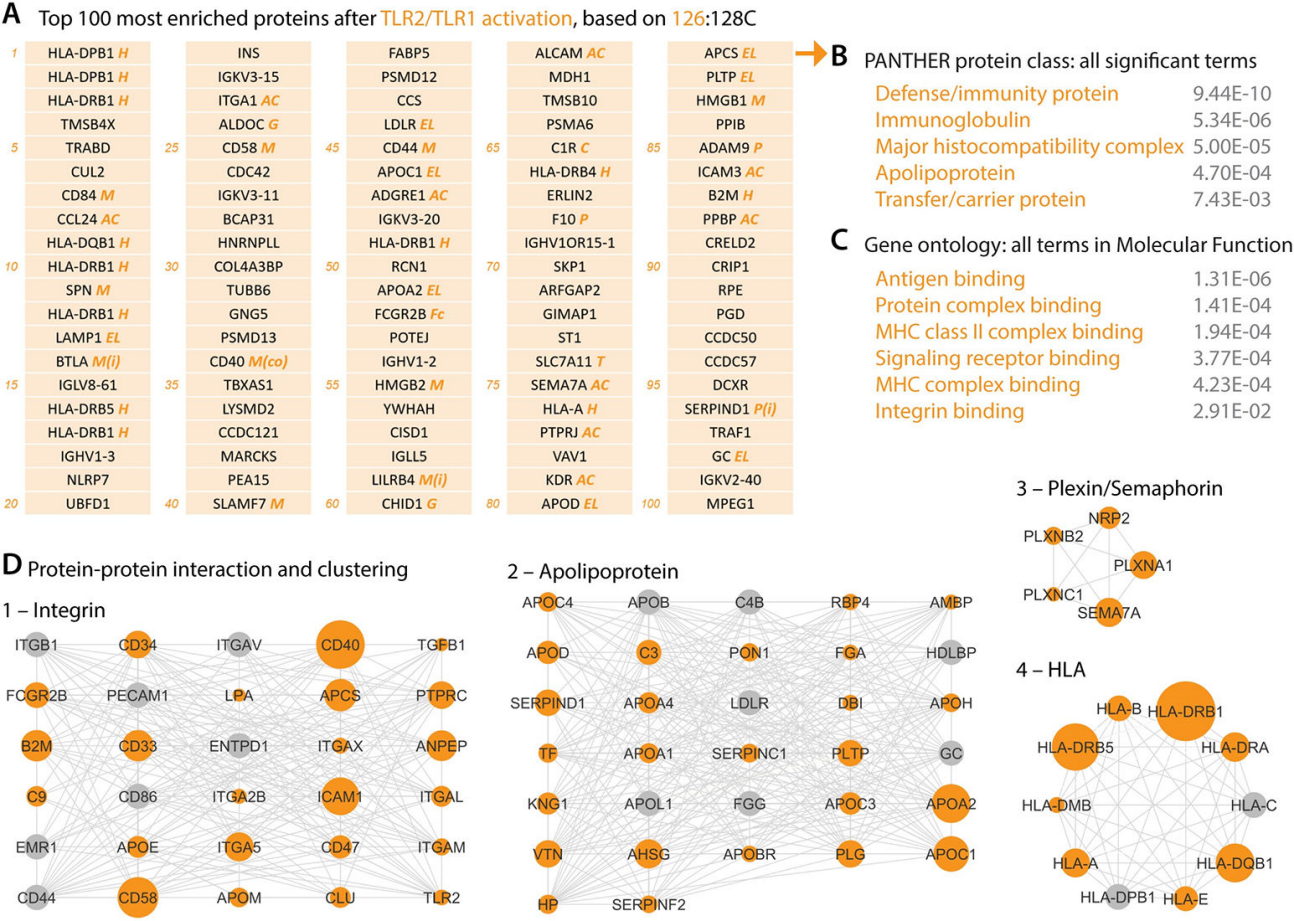
Cell-surface milieu and protein networks of TLR2/TLR1-activated human cDCs. (**A**) Top 100 most enriched proteins of activated cDCs, ranked by the TMT ratio 126:128C. Italicized marks annotate protein families and functions: H, human leukocyte antigens (HLAs); M, immune modulation molecules, including co-stimulatory (co) and inhibitory (i) signals; EL, endocytosis and lysosome-related; AC, adhesion and chemotaxis including integrins; P, proteases, peptidases, and their inhibitors (i); C, complement system; Fc, Fc receptors; G, glycosylation; and T, transporters. Due to HLA protein similarity and polymorphism, unambiguously assigning HLA alleles with tryptic peptides without paired DNA sequencing is technically challenging (24). Some HLA proteins, such as HLA-DPB1 and HLA-DRB1, were thus called and listed repeatedly by the search algorithm. See Supplementary Files for their UniProt accession numbers. (**B** and **C**) Functional classification of the top 100 most enriched proteins of activated cDCs (listed in **A**) by Panther (**B**) and gene ontology (**C**). Gray numbers indicate the false discovery rate of each term. (**D**) Protein-protein interaction informed clusters of activated cDC cell-surface proteins. Dot size indicates the enrichment extent based on the TMT ratio 126:128C. Gray dots were not detected in our proteomic experiment but added by Cytoscape in clustering analysis.

To identify the most enriched proteins on the cDC cell surface, we adopted a TMT-based ratiometric strategy (28) in which the TMT ratio of each protein reflects its differential enrichment in the cell-surface biotinylation group (e.g., 126 in Fig. 2B) versus the non-biotinylation control group (e.g., 128C in Fig. 2B). By ranking the proteome (Supplemental Table – Tab 3) with the 126:128C TMT ratio (Fig. 2B), we found that many class I and class II HLA proteins (Fig. 3A, marked as ‘H’, and Fig. 3D_4_), as well as the co-stimulatory molecule CD40 (34^th^ in Fig. 3A), were among the top 100 most enriched proteins on the activated cDC cell surface but not the resting cDC cell surface (ranked by 129N:127N; Supplemental Fig. 2A), validating the proteomic data by these known hallmarks of cDC activation.

The top 100 cell-surface proteins of activated cDCs highlighted several molecular families that play central roles in immunity (Fig. 3B, 3C): the antigen presentation machinery class I/II HLA proteins (marked as ‘H’ in Fig. 3A), immune modulatory molecules including co-stimulatory and inhibitory signals (‘M’ in Fig. 3A), endocytosis and lysosome proteins (‘EL’ in Fig. 3A), and adhesion and chemotaxis molecules (‘AC’ in Fig. 3A), constituting a profoundly different cell-surface milieu compared to the resting cDCs (Supplemental Fig. 2A). In addition, immunoglobulin proteins (IGHV, IGLV, and IGKV) were abundant on the activated cDC surface (Fig. 3A), likely due to the binding of antibodies to cDC surface Fc receptors.

Cell-surface proteins of activated cDCs formed functionally-associated networks through protein-protein interactions (Fig. 3D), with one cluster of apolipoproteins shared with the resting cDCs (Fig. 3D_2_, Supplemental Fig. 2B) suggesting a housekeeping role of apolipoprotein-mediated transport in DC physiology across cell states. Protein interaction based clustering highlighted several Plexin and Semaphorin proteins (Fig. 3D_3_), which are evolutionarily conserved axon guidance factors in brain development (29). Intriguingly, other families of axon guidance molecules (30, 31) were not seen on the cDC surface, suggesting unique properties of the Plexin-Semaphorin signaling axis in cDC function (32).

### Coordinated remodeling creates a pro-inflammatory cell-surface milieu

**Figure 4.**
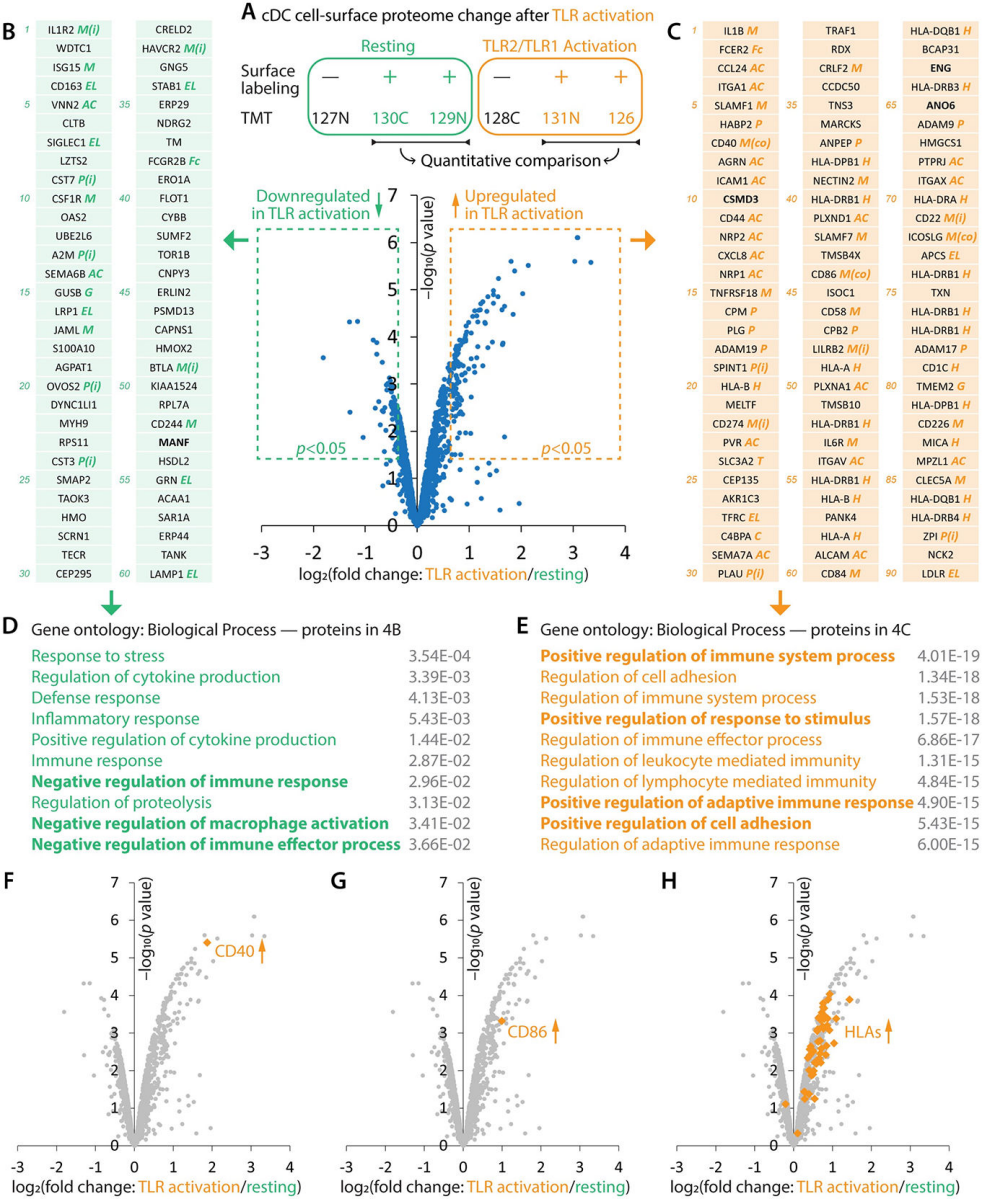
Activation globally remodels the cDC cell-surface proteome. (**A**) TMT-based quantitative comparison revealed the expression change of cell-surface proteins in cDC activation. (**B** and **C**) Sixty most down-regulated (**B**; log_2_ fold change<−0.385 and *p*<0.05) and ninety most up-regulated (**C**; log_2_ fold change>0.753 and *p*<0.05) cDC cell-surface proteins. Italicized marks annotate protein families and functions: H, human leukocyte antigens (HLAs); M, modulation including co-stimulatory (co) and inhibitory (i) signals; EL, endocytosis and lysosome-related; AC, adhesion and chemotaxis including integrins; P, proteases, peptidases, and their inhibitors (i); C, complement system; Fc, Fc receptors; G, glycosylation; and T, transporters. (**D** and **E**) Functional gene ontology analyses for the most down- (**D**) and up-regulated (**E**) proteins. (**F**–**H**) Upregulation of known cDC activation marker proteins CD40 (**F**), CD86 (**G**), and HLAs (**H**).

Besides examining the activated and resting cDC cell-surface proteomes separately (Fig. 3, Supplemental Fig. 2, Supplemental Table – Tab 3), TMT-based profiling also allowed us to directly and quantitatively measure the change of each individual protein in cDC activation (Fig. 4, Supplemental Table – Tab 4). Consistent with the observation that activated and resting cDCs had distinct cell-surface proteomic landscapes (Fig. 3A, Supplemental Fig. 2A), more than one hundred proteins exhibited significant up- or down-regulation upon cDC activation (Fig. 4A-C, Supplemental Table – Tab 4). Known cDC activation markers, such as CD40 (Fig. 4F, 7^th^ in Fig. 4C), CD86 (Fig. 4G, 44^th^ in Fig. 4C), and HLAs (Fig. 4H, marked as ‘H’ in Fig. 4C), were among the most up-regulated proteins (Fig. 4C).

**Figure 5.**
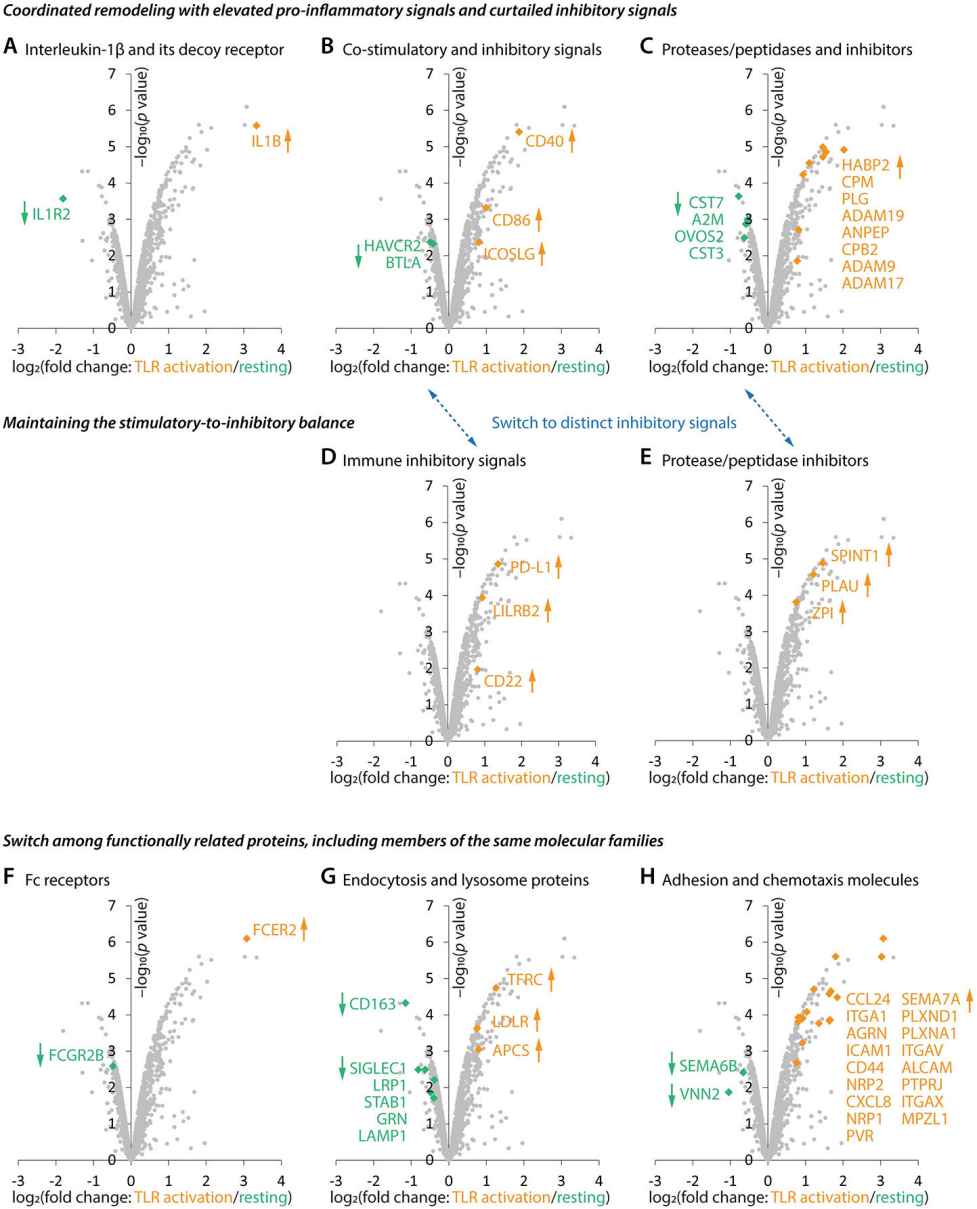
Molecular and organizational features of cDC cell-surface remodeling in activation. (**A**–**C**) Across multiple molecular families, including interleukin (**A**), immune modulation (**B**), and protein homeostasis (**C**), the cDC cell-surface remodeling is coordinated to concurrently escalate immune effectors while down-regulating inhibitory signals. (**D** and **E**) Activated cDCs leverage distinct inhibitory molecules for immune modulation (**D**) and protein homeostasis (**E**). (**F**–**H**) Functionally related cell-surface proteins, including molecular ‘siblings’ from the same families, are swapped in cDC activation.

Functional gene ontology analysis of the ninety most up-regulated proteins (Fig. 4C) yielded uniform terms of positive regulation of immunity (Fig. 4E) while the sixty most down-regulated proteins (Fig. 4B) enriched terms of negative regulation of immunity (Fig. 4D), highlighting the coordination in cDC cell surface remodeling: escalating positive drivers while simultaneously curtailing negative brakes to promote immune responses. This coordination was observed across several protein families:

1) The most up-regulated protein was interleukin-1 beta (IL1B; Fig. 5A, 1^st^ in Fig. 4C), a potent pro-inflammatory cytokine. The most down-regulated protein was interleukin-1 receptor type 2 (IL1R2; Fig. 5A, 1^st^ in Fig. 4B), a non-signaling decoy receptor that reduces IL1B activity by competing with the signaling receptor IL1R1 (33).

2) Several co-stimulatory molecules, such as CD40 (7^th^ in Fig. 4C), CD86 (44^th^ in Fig. 4C), and ICOSLG (72^nd^ in Fig. 4C) (34), went up while potent inhibitory signals—HAVCR2 (32^nd^ in Fig. 4B) and BTLA (49^th^ in Fig. 4B) (35, 36)—went down (Fig. 5B).

3) It is a salient feature that activated cDCs elevated many proteases and peptidases (Fig. 5C, marked as ‘P’ in Fig. 4C), likely linked to cell migration, tissue remodeling, and cell-surface proteome remodeling (37). Concurrently, cDCs also reduced several protease and peptidase inhibitors (Fig. 5C, marked as ‘P(i)’ in Fig. 4B).

### Elevating select inhibitory signals for stimulatory-to-inhibitory balance

Although coordination can enable fast and potent immune responses, it also poses a threat of overreaction. Fig. 4C, Fig. 5D, and Fig. 5E show that activated cDCs leveraged a hedging strategy to maintain the stimulatory-to-inhibitory balance: 1) While elevating positive immune modulators, cDCs also up-regulated inhibitory signals (Fig. 5D), including PD-L1 (CD274; 22^nd^ in Fig. 4C) and LILRB2 (48^th^ in Fig. 4C) (38, 39). 2) Several protease and peptidase inhibitors (Fig. 5E, marked as ‘P(i)’ in Fig. 4C) went up along with their targets of inhibition. Thus, activated cDCs switched their immune inhibitory signals from HAVCR2/BTLA (‘M(i)’ in Fig. 4B) to PD-L1/LILRB2 (‘M(i)’ in Fig. 4C) and also switched to a distinct collection of protease and peptidase inhibitors (‘P(i)’ in Fig. 4B, 4C; see also Fig. 5B vs. 5D and Fig. 5C vs. 5E).

This phenomenon of switching among functionally related molecules extended beyond inhibitory proteins: 1) Fc receptor (Fig. 5F) from FCGR2B (38^th^ in Fig. 4B) to FCER2 (2^nd^ in Fig. 4C); 2) endocytosis and lysosome proteins (Fig. 5G, ‘EL’ in Fig. 4B, 4C); and 3) adhesion and chemotaxis molecules (Fig. 5H, ‘AC’ in Fig. 4B, 4C), including the switch of Semaphorin from SEMA6B (14^th^ in Fig. 4B) to closely related SEMA7A (29^th^ in Fig. 4C). Therefore, although more than one hundred proteins were elevated or reduced, the global remodeling of the cDC cell-surface proteome was not coarse but rather finely tuned, including exchanges of ‘sibling’ proteins within each molecular family.

## DISCUSSION

### cDC states and cell-surface proteome dynamics

Here, we present the cell-surface proteomes of human primary cDCs in resting and TLR2/TLR1-activated states. Quantitative mass spectrometry found that, within 18 hours after TLR activation, the cDC cell-surface underwent a global remodeling instead of changing only a few molecules. By simultaneously elevating pro-inflammatory signals and subsiding inhibitory signals, activated cDCs leverage a coordinated program to promote immune responses. Meanwhile, a hedging strategy employing a different set of inhibitory signals is implemented to maintain the stimulatory-to-inhibitory balance.

Numerous innate immune receptors are expressed by cDCs—TLR2/TLR1 is only one pair of the many. Distinct receptors are known to use very different signaling pathways upon activation (40), thus likely conferring cDCs with different states and unique functional outcomes. This study provides a scalable approach for systematically examining whether activating distinct receptors remodels the cDC cell-surface proteome in different ways and leverage different effector molecules, which may help understand why distinct infections lead to drastically different immune responses. Furthermore, higher resolution profiling of cDC subtypes as well as plasmacytoid DCs (7, 8, 41) may shed light on subtype-specific cell-surface protein composition and dynamics.

We also note that the current method—Sulfo-NHS-mediated biotinylation followed by enrichment of biotinylated proteins with streptavidin—has a technical caveat of capturing certain intracellular contaminants (mainly, non-specific proteins that bind to streptavidin beads and get eluted by on-bead trypsin digestion, endogenously biotinylated proteins, and intracellularly biotinylated proteins in dead cells) and requires filtering in data analysis. Future chemical tool development enabling bio-orthogonal labeling of cell-surface proteins with higher spatial specificity in human primary cells may further empower the study of cell-surface milieu in immunity.

### Opportunities and challenges in a causal understanding of the cDC cell-surface proteome

Our data provides a resource and roadmap for exploring the cDC cell-surface milieu (Fig. 3A, 4B, 4C, Supplemental Fig. 2A, Supplemental Table – Tab 1, 3, 4). For instance, several distinct inhibitory molecules, such as CD274 (PD-L1) and CD22, were selectively up-regulated in cDC activation (Fig. 4C), suggesting their prospective roles in signal thresholding in cDC-lymphocyte interactions. In Fig. 4B and 4C, we highlighted a few intriguing molecules (bold) without any known functions in the immune system: MANF (53^rd^ in Fig. 4B), a neurotrophic factor (42); CSMD3 (10^th^ in Fig. 4C), an unstudied transmembrane protein in any system; ENG (63^rd^ in Fig. 4C), an angiogenesis factor (43); and ANO6 (65^th^ in Fig. 4C), a calcium-activated cation channel and phospholipid scramblase (44). Further genetic and causal studies may reveal whether and how they participate in TLR2/TLR1-mediated cDC activation.

On the other hand, the data also poses a profound challenge. As listed in Fig. 4B and 4C, more than one hundred proteins were down- or up-regulated. Many of them have apparent immune functions, either studied or predictable by their molecular families. Moreover, they form complex protein-protein interactions (Fig. 3D). How would such a large cohort of proteins collectively contribute to the various functions of activated cDCs? Classical single-gene interrogation will likely be interfered by redundancy and network compensation. Technological development of highly-multiplexed gene or protein manipulation, along with content-rich assays, would be instrumental for examining the combinatorial effect of many molecules and establishing functional causality of this and many other omics data, especially for the study of human biology (45).

## Supplementary Material

1

2

## KEY POINTS

Cell-surface proteome of human primary conventional dendritic cells

Global and coordinated remodeling of the cell-surface milieu in cDC activation

Stimulatory-to-inhibitory balance for immune modulation and protease activity
