# Supplementary material for: Cell-surface milieu remodeling in human dendritic cell activation
Source: J Immunol. Author manuscript; Available in PMC 2024 Oct 1. (PMC11408084; doi:10.4049/jimmunol.2400089)
Supplement: 1 [file NIHMS2013126-supplement-1.docx]

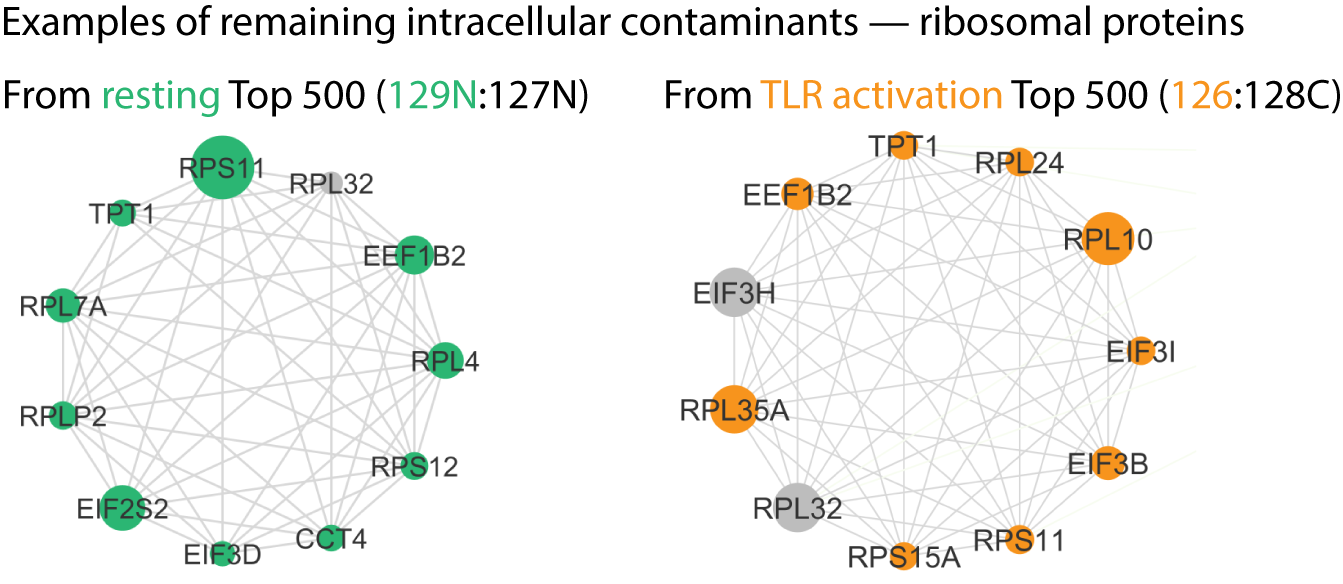


**Supplemental Figure 1.** Examples of remaining intracellular contaminants after filtering shown in **Fig. 2E**.

**
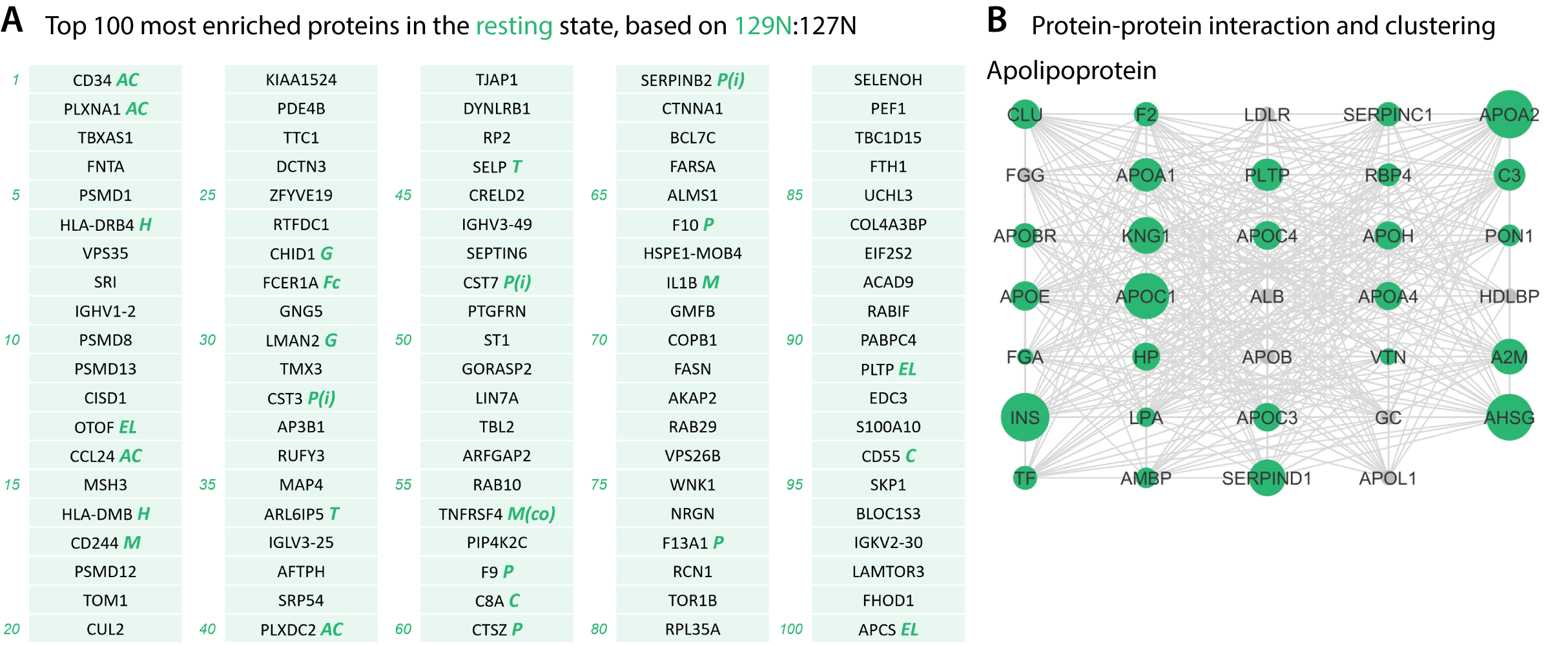
**

**Supplemental Figure 2.** Cell-surface proteome of resting human cDCs.

(**A**) Top 100 most enriched proteins of resting cDCs, ranked by the TMT ratio 129N:127N. Italicized marks annotate protein families and functions: H, human leukocyte antigens (HLAs); M, modulation including co-stimulatory (co) and inhibitory (i) signals; EL, endocytosis and lysosome-related; AC, adhesion and chemotaxis including integrins; P, proteases, peptidases, and their inhibitors (i); C, complement system; Fc, Fc receptors; G, glycosylation; and T, transporters. We note that more intracellular contaminants are seen on this list compared with **Fig. 3A**, likely because unstimulated cDCs are more prone to cell death and consequent intracellular labeling by Sulfo-NHS.

(**B**) A protein-protein interaction informed cluster of resting cDC cell-surface proteins. Dot size indicates the enrichment extent based on the TMT ratio 129N:127N. Gray dots were not detected in our proteomic experiment but added by Cytoscape in clustering analysis.
